# Construct validity of a dietary protein assessment questionnaire to explore college students’ knowledge and attitudes towards dietary protein

**DOI:** 10.3389/fnut.2023.1289946

**Published:** 2023-12-07

**Authors:** Parker Gutjahr, Cynthia Warren, Derek Miketinas

**Affiliations:** ^1^Department of Nutrition and Food Sciences, Texas Woman’s University, Denton, TX, United States; ^2^Department of Nutrition and Food Sciences, Texas Woman’s University, Houston, TX, United States

**Keywords:** questionnaire, knowledge-attitude-behavior, dietary protein, factor analysis, statistical approaches, college students

## Abstract

**Introduction:**

Misconceptions about dietary protein may exist due to unscientific information from commonly used sources such as social media. Understanding knowledge and attitudes towards protein is important for developing effective interventions to improve the dietary behaviors of U.S. college students. The objective of this study was to develop a questionnaire to evaluate college students’ knowledge and attitudes towards dietary protein.

**Methods:**

The questionnaire had 64 questions, including 8 demographic, 24 knowledge, 14 attitude, and 18 behavior questions. Construct validity of the knowledge questions was assessed by performing known-group comparisons using an independent t-test. Exploratory factor analysis (EFA) with principal axis factoring and a promax rotation was used to evaluate the factor structure of the attitude questions.

**Results:**

Four hundred seventy participants (87.3% female) provided responses for the attitude questions. Fifty-five nutrition and Fifty-one non-nutrition students provided responses for the knowledge questions. Three factors were retained: animal protein sources’ relationship with human and environmental health (Factor 1); organic protein sources (Factor 2); and adequacy of the protein recommended dietary allowance (RDA) for weight loss and vegetarian diets (Factor 3). Mean knowledge responses were 66.4 ± 11.5% and 47.6 ± 16.4% for nutrition and non-nutrition students, respectively (*t*-test *p*-value for difference <0.001).

**Conclusion:**

Protein attitudes appear multidimensional and correlated. Further testing is needed to confirm the three-factor model and to assess temporal reliability.

## 1 Introduction

Protein is a major structural and functional component of the human body, accounting for approximately 14%–16% to the total mass of a lean adult (1, 2). Dietary protein intake recommendations vary by life cycle phase, disease state, and physical activity (2–6). The Recommended Dietary Allowance (RDA) for protein for healthy adult men and women is 0.8 grams per kilogram of body weight per day (g/kg/day), which is based on careful analyses of available nitrogen balance studies (2). Dietary protein recommendations vary across professional organizations, such as the International Society of Sports Nutrition, Academy of Nutrition and Dietetics, and Institute of Medicine, based on physical activity and age (2, 3, 5). The International Society of Sports Nutrition recommends 1.4–2.0 g/kg/day for an athlete who lifts weights or is training for an endurance event (3). The Academy of Nutrition and Dietetics recommends that a protein intake of 1.0–1.6 g/kg/day for older adults >60 years is safe and adequate to meet their needs, while the Institute of Medicine suggests older adults do not have elevated protein needs above 0.8 g/kg/day (2, 5). The Acceptable Macronutrient Distribution Range (AMDR) for protein varies by age and is 10%–35% of total calories for adults >18 years (2). These discrepancies are of consequence for health professionals who provide dietary recommendations for patients and for young adults and athletes who seek recommendations from reputable sources.

The average protein intake in the United States (U.S.) is close to the *Dietary Guidelines for Americans’* recommendations for all age-sex groups; however, the average intake of different protein sources vary in comparison to the recommendations, especially for seafood (7). These dietary behaviors may be due to poor nutrition knowledge, poor attitudes towards food and nutrition, use of unreliable sources, lack of availability and/or accessibility to food sources, and/or unawareness of evidence-based recommendations (8, 9). Dietary behaviors are influenced by many factors including nutrition knowledge and attitudes towards food and nutrition (8–10). Attitudes towards food and nutrition are formed in part by nutrition knowledge. Greater nutrition knowledge has been associated with positive attitudes towards food and nutrition, as well as increased adherence to dietary recommendations (8–10). Unhealthy dietary behaviors among college students, such as high intakes of fast food and low intakes of fruits and vegetables, have been observed (11, 12). Additionally, lack of knowledge about protein has been found among college students, despite common use of protein supplements (13). Low levels of nutrition knowledge, as well as poor attitudes towards protein, may be due to unsubstantiated nutrition information (14).

There is limited research available on protein knowledge and attitudes among U.S. college students; and no validated instrument exists to accurately assess these constructs (9). It is crucial to understand protein knowledge and attitudes to design and implement appropriate education tools, increase awareness, and address misconceptions. Considering these limitations, the Dietary Protein Assessment Questionnaire (DPAQ) is under development to quantify dietary protein knowledge, attitudes, and sources of nutrition information so that researchers can explore the relationships between these constructs and outcomes. The DPAQ will ultimately help professionals create and provide appropriate educational interventions and resources to help improve the health of U.S. college students. This study provides valuable preliminary data on construct validity of the knowledge and attitude questions, which will guide future development of the DPAQ to become the first validated instrument for dietary protein.

## 2 Materials and methods

### 2.1 Item generation

The items for the DPAQ were generated using principles from Don Dillman’s book on survey development (15). The DPAQ consisted of 64 questions on the knowledge, attitudes, and behaviors towards protein, including 8 demographic questions. The knowledge questions consisted of three answer choices (true, false, unsure) and were created to assess respondents’ knowledge about dietary protein sources and requirements for various populations, such as physically active individuals and individuals adhering to a vegetarian diet. The attitude questions included a 5-point Likert scale ranging from “strongly disagree = 1” to “strongly agree = 5” with a neutral midpoint to assess respondents’ attitudes towards plant and animal protein sources. The behavior questions consisted of multiple-choice answer options to assess respondents’ dietary patterns regarding protein.

Nutritional science researchers reviewed the questionnaire for applicability, structure, reading level, and comprehension. The questionnaire was then updated according to feedback. Cognitive interviews were conducted using individuals with no nutrition background to assess information-processing needs of the questionnaire items (16). Researchers and statisticians reviewed the questionnaire to identify appropriate scaling of answer choices and the questionnaire was updated to create the final version prior to distribution. The DPAQ was then administered using PsychData (PsychData.com, LLC, State College, PA). See Supplementary material for the version of the DPAQ that was administered.

### 2.2 Sample and recruitment

In the fall 2018, participants were recruited through an open call email sent to students attending Texas Woman’s University. The email informed potential participants of the study’s purpose, eligibility requirements, and included a link to the DPAQ. Participants were recruited with the help of professors and researchers to voluntarily complete the questionnaire. The online questionnaire link was also posted on social media sites and spread by word of mouth. Eligibility requirements included individuals ≥18 years of age with a reliable Internet source.

Data were collected from nutrition undergraduate students enrolled in a junior-level nutrition class and from non-nutrition undergraduate students enrolled in a junior-level education class as a comparison group for the knowledge section. Students were offered extra credit in their respective classes for successful completion of the questionnaire.

Approval of the study was obtained from Texas Woman’s University Institutional Review Board. Informed consent was collected from each participant before participation in the questionnaire. Data were de-identified except for the nutrition and education students used for the knowledge section.

### 2.3 Validity measures and data analyses

For the attitude questions, participants were randomly partitioned into two analytic samples. One sample was used to identify possible factor structures, while the other was used to re-evaluate the factor structure. The correlation matrix and factor loading scores for both analytic samples were examined, and items were eliminated according to criteria.

Exploratory factor analysis (EFA) with principal axis factoring and a promax rotation was performed on the 14 attitude questions to identify the dimensionality of the attitude constructs for the subjects. The correlation matrix was examined for items exhibiting multicollinearity (*r* ≥ 0.9). Factor retention criteria included factors ≥|0.4| and factors comprised of two or more items. Composite scores for the factors were calculated according to their factor loadings. Internal reliability was examined using Cronbach’s *α*. The questionnaire responses were then compared across gender, education, and race/ethnicity using an ANOVA and adjusted for multiple comparisons using the Tukey–Kramer adjustment where necessary.

The knowledge questions were evaluated for construct validity by comparing mean scores between nutrition and non-nutrition majors using independent samples *t*-test. The correct answers were totaled for each student to determine the mean scores. The answers marked “unknown” were given a value of zero and did not contribute to overall mean scores. A *p* < 0.05 was considered statistically significant for all analyses unless otherwise indicated. All data analysis was performed with SAS^®^ software, Version 9.4 Statistical Analysis System (RRID:SCR_008567). Copyright^©^ 2013 SAS Institute Inc. SAS and all other SAS Institute Inc. product or service names are registered trademarks or trademarks of SAS Institute Inc., Cary, NC, United States.

## 3 Results

### 3.1 Questionnaire participants

Four hundred seventy responses were received and 450 provided complete demographic data. Most participants were female (87.3%), and the mean age was 28.2 ± 11.4y. See Table 1 for complete demographic information.

**Table 1:** Demographic data for questionnaire participants randomized to exploratory factor analysis.

Variable	Total sample (*n* = 450)	EFA #1 (*n* = 225)	EFA #2 (*n* = 225)	*p*
**Age** (mean ± SD)	28 ± 11.4	28 ± 11.7	29 ± 11.0	0.37
**Sex *n* (%)**				
Female	393 (87)	205 (91)	188 (84)	0.02^*^
**Race *n* (%)**				
Caucasian	294 (65)	148 (66)	146 (65)	0.92
Hispanic	77 (17)	34 (15)	43 (19)	0.32
African American	55 (12)	28 (12)	27 (12)	1.00
Asian/Pacific Islander	34 (8)	19 (8)	15 (7)	0.59
American Indian	13 (3)	6 (3)	7 (3)	1.00
Other	7 (2)	2 (1)	5 (2)	0.45
**Health status *n* (%)**				
Healthy	356 (79)	182 (81)	174 (77)	0.42
Overweight/obese	117 (26)	57 (25)	60 (27)	0.83
Diabetes	7 (2)	3 (1)	4 (2)	1.00
High cholesterol	26 (6)	11 (5)	15 (7)	0.55
CKD	3 (1)	2 (1)	1 (<1)	1.00
High blood pressure	16 (4)	5 (2)	11 (5)	0.20
**Education *n* (%)**				0.59
High school	57 (13)	31 (14)	26 (12)	
Some college	84 (19)	47 (21)	37 (16)	
Associate degree	66 (15)	29 (13)	37 (16)	
Bachelor’s degree	153 (34)	75 (33)	78 (35)	
Graduate degree	90 (20)	43 (19)	47 (21)	

CKD, chronic kidney disease; ^*^p < 0.05; group difference for the continuous variable was assessed using the independent *t*-test; group differences for categorical variables were assessed using chi-square test for independence.

### 3.2 Exploratory factor analysis #1

Two hundred twenty-five participants were randomized to the first EFA; 74.2% provided complete responses for the attitude questions. The data exhibited good sampling adequacy (Kaiser–Meyer–Olkin test = 0.8) and the correlation matrix was suitable for structure detection (Bartlett’s test <0.001). There was some evidence of multicollinearity observed within the correlation matrix (determinant = 0.006). A total of five items did not meet inclusion criteria (primary factor loading ≥|0.4|) and were removed for the subsequent EFA. The first EFA retained four factors and explained 62.3% of the total variance.

### 3.3 Exploratory factor analysis #2

Two hundred twenty-five respondents were randomized to the second EFA; 59.1% provided complete responses for the attitude questions. The data demonstrated good sampling adequacy (Kaiser–Meyer–Olkin test = 0.76) and the correlation matrix was suitable for structure detection (Bartlett’s test <0.001). The correlation matrix was examined for items exhibiting extreme multicollinearity (determinant = 0.007). There was some evidence of multicollinearity observed among the statements of “meat consumption is unhealthy” and “meat should not be consumed” (*r* = −90). Three factors were retained, which were comprised of the nine items remaining from EFA #1 and explained 73.9% of the total variance. See Table 2 for variance explained by each factor. All items displayed a factor loading ≥|0.4|.

**Table 2:** Exploratory factor analysis pattern and structure matrices with communalities and explained variance by factor (*n* = 225).

Items by factor	Pattern matrix	*h^2^*	Structure matrix	Explained variance
**Factor 1: human/environmental health**				44.6%
The impact of climate change can be reduced by consuming less meat, dairy, & eggs	0.74	0.49	0.69	
Meat production is harmful to the environment	0.85	0.63	0.79	
Egg consumption is harmful to human health	−0.54	0.63	−0.61	
Meat consumption is unhealthy	−0.88	0.81	−0.90	
Meat should not be consumed	0.81	0.76	0.87	
**Factor 2: organic sources**				15.5%
Organic protein sources are better for the environment	0.67	0.59	0.75	
Organic protein sources are healthier	1.01	0.92	0.95	
**Factor 3: protein RDA**				13.8%
The RDA for protein is adequate for healthy weight loss	0.59	0.33	0.57	
The RDA for protein is adequate for people following a vegetarian diet	0.84	0.72	0.85	

RDA, recommended dietary allowance (for protein: 0.8 g/kg/day); *h*^2^ denotes the communalities.

Factor 1 included five items related to animal protein sources and their relationship with human and environmental health. Factor 2 included two items pertaining to the healthfulness of organic protein sources. Factor 3 included two items describing the adequacy of the RDA for protein with respect to weight loss and adherence to a vegetarian diet. Factor 1 shared a moderate, inverse relationship with Factor 2 (*r* = −0.47), and a weak, positive relationship with Factor 3 (*r* = 0.29). Factor 2 shared a weak, inverse relationship with Factor 3 (*r* = −0.19). Cronbach’s α coefficient for Factor 1 (*α* = 0.87) and Factor 2 (*α* = 0.83) displayed evidence of good internal reliability. Satisfactory internal reliability was observed for Factor 3 (*α* = 0.65).

### 3.4 Knowledge towards protein

Fifty-five nutrition undergraduate students and 51 education undergraduate students’ responses were analyzed. The majority of participants were female (95.3%) and the mean age was 27.9 ± 11.2 years. The nutrition students’ mean test score was 66.4 ± 11.5% with scores ranging from 42%–92%. The education students’ mean test score was 47.6 ± 16.4% with scores ranging from 17%–79%. A significant difference in mean test score values was observed between nutrition and education students (18.8 ± 14.1; *p* < 0.001). Cohen’s *d* indicated a large, standardized difference between nutrition and education mean scores (*d* = 1.33).

## 4 Discussion

Currently, no validated questionnaires exist that attempt to measure the knowledge and attitude constructs of protein among the college student population (17, 18). As a result, studies that evaluated knowledge and attitudes towards specific macronutrients lacked validated instruments (17–23). This study provided evidence of construct validity for the DPAQ’s protein knowledge and attitudes.

The EFA identified a multidimensional structure, and the original 14 attitude items could be shortened by five items without decreasing internal reliability. Five items loaded strongly with human/environmental health (Factor 1). Items contributing positively to the Factor 1 score included “meat production is harmful to the environment,” “meat should not be consumed,” and “the impact of climate change can be reduced by consuming less meat, dairy products, and eggs.” Items contributing negatively included “meat consumption is unhealthy” and “egg consumption is harmful to human health.” The inverse contributions of the items “meat consumption is unhealthy” and “meat should not be consumed” to the overall Factor 1 score suggest that college students’ consider environmental health more than human health when determining food items that should and should not be consumed. This could be a misconception among college students due to social media platforms being one of their main sources of nutrition and health information.

Two items loaded strongly with organic sources (Factor 2), which suggests that college students believe that organic protein sources are healthier to consume and better for the environment compared to conventional (non-organic) protein sources. Although the exact extent is unknown, this shows that college students place some value on organic protein sources. Two items also loaded strongly with protein RDA (Factor 3), which shows that college students believe the RDA for protein is adequate in terms of healthy weight loss and people adhering to a vegetarian diet. The factor structure provides evidence that attitude constructs towards protein are multidimensional.

Future development of the DPAQ should further develop the attitude constructs. An analysis of the relationship between nutrition information sources and the attitude constructs would be beneficial to identify strategies to educate college students. Adding more items related to Factors 2 and 3 may help define the factors and may strengthen the correlations observed among the protein attitudes measured.

The significant difference in mean test scores between the undergraduate nutrition and non-nutrition (education) students indicated that the DPAQ instrument had adequate construct validity. The nutrition students’ mean test score was greater than the non-nutrition students, which has been observed in previous studies (23–27). The mean test score of nutrition students in the current study was lower than those in previous studies, which may be due to many factors, such as administering the questionnaire without prior notice or wording of knowledge statements (19–22). It is important to note the instruments used in previous studies had content not exclusively on protein, but included content related to general nutrition and salt knowledge among adult and student populations (18–22).

While studies have shown dietary patterns can be influenced by eating motives and the perceived impacts on human health and the environment, more research is needed (18–22, 28). With further development, the DPAQ may be used to identify knowledge and attitudes towards protein on the topics of human/environmental health, organic sources, and adequacy of the RDA, as well as other topics needed to capture the full nature of protein attitudes.

Due to increased popularity of social media platforms, there has been a commensurate rise in the amount of false nutrition information presented to the public (29–31). The lack of “media literacy” may contribute to this wide range of false information. Therefore, it is necessary to create validated instruments to assess protein attitudes and knowledge among the public. Identifying protein knowledge and attitudes will facilitate the design and development of education tools to increase awareness and decrease misconceptions currently associated with protein. Interventions targeting various factors, such as eating motives and reliable nutrition sources, may also lead to improved understanding of evidence-based protein intake.

The strengths of this study include sample sizes, internal consistency of items, and utilizing the evidence-based approach for questionnaire development; however, several limitations exist. Although participants were homogenous in gender, age, and race, results may not be generalizable to other populations. It is important to examine validity in a more diverse population before conducting broader population studies. Just like any self-reported item, this study is also limited by the truthfulness of participants. Satisfactory internal reliability (*α* ≤ 0.70) was identified for Factor 3, which may provide evidence of inconsistent answers to attitude questions regarding protein RDA (32). Future studies should focus on increasing internal reliability of the DPAQ by adjusting the number of items, rewording questions, and reformatting the instrument. The instrument’s validity should be examined in a more diverse population with a more equal gender distribution to increase generalizability to the college student population, as well as provide more complex measurements to explore the attitude constructs multidimensionality.

## 5 Conclusion

The results of this study provide preliminary evidence for the knowledge and attitude constructs validity within the DPAQ to be used among the college student population. The instrument, and, in particular, the topic on “protein RDA” requires further development. Attitudes towards protein seem multidimensional and correlated. Additional testing is needed on the DPAQ to confirm the three-factor model and to estimate test-retest reliability. A multidimensional approach seems crucial for future development of the DPAQ, as well as for effective interventions. Future development should focus on increasing internal reliability by adjusting the number of items, rewording questions, and reformatting the instrument. This will allow the DPAQ to be administered to more diverse populations, which will enable researchers to accurately measure protein knowledge and attitudes to create effective nutrition interventions for college students.

## Data availability statement

The raw data supporting the conclusions of this article will be made available by the authors, without undue reservation.

## Ethics statement

The studies involving humans were approved by Texas Woman’s University Institutional Review Board. The studies were conducted in accordance with the local legislation and institutional requirements. The ethics committee/institutional review board waived the requirement of written informed consent for participation from the participants or the participants’ legal guardians/next of kin because this study utilized an online questionnaire, so it was not possible to obtain a signature from participants. Informed consent was obtained from participants by providing the consent form on the first page of the questionnaire. Since this study was an exempt study involving a questionnaire, a complete consent form was not required. The following statement, which was required, appeared on the consent form instead: the completion of this questionnaire constitutes your informed consent to act as a participant in this research.

## Author contributions

PG: Visualization, Conceptualization, Formal analysis, Investigation, Visualization, Writing – original draft. CW: Methodology, Resources, Supervision, Visualization, Writing – review & editing. DM: Visualization, Data curation, Conceptualization, Formal analysis, Methodology, Resources, Supervision, Visualization, Writing – review & editing.
