# Supplementary material for: Construct validity of a dietary protein assessment questionnaire to explore college students’ knowledge and attitudes towards dietary protein
Source: Front Nutr. 2023 Dec 7;10:1289946. doi: 10.3389/fnut.2023.1289946 (PMC10733519; doi:10.3389/fnut.2023.1289946)
Supplement: Supplementary file 1 [file Data_Sheet_1.PDF]

Dietary Protein Assessment Survey

Demographics

1) What is your age?

2) Please indicate your gender.
- Male
- Female

3) Please indicate your ethnicity (Choose all that apply).
- African American
- American Indian
- Asian/Pacific Islander
- Caucasian (non-Hispanic)
- Hispanic
- Scandinavian
- Prefer not to disclose
- Other (please specify)

4) What best describes your lifestyle environment?
- Urban (city)
- Suburban (small residential community)
- Rural (country)

5) What is your current health status (Choose all that apply)?
- Healthy
- Overweight/Obese
- Diabetic
- High blood pressure
- High cholesterol
- Chronic Kidney Disease
- Prefer not to disclose
- Other (please specify)

6) What is the highest level of education you have attained?
- Less than a high school diploma
- High school graduate
- Some college or technical training
- Associate's degree or equivalent
- Bachelor's degree
- Graduate degree

Under Development

7) What is your current work situation?
- Employed full-time
- Employed part-time
- Semi-retired
- Fully retired
- Self-employed
- Unemployed
- Homemaker
- Student
- On disability
- Other (please specify)

8) What is your total combined household income for the past year?
- Less than $5,000
- $5,000 - $19,000
- $20,000 - $49,000
- $50,000 - $99,999
- $100,000 - $150,000
- More than $150,000
- Prefer not to disclose

Knowledge

9) The Recommended Dietary Allowance (RDA) for protein for a healthy male or female adult is 0.8 grams per kilogram of body weight per day.
- True
- False
- Unsure

10) Dairy products (milk, cheese, yogurt) are a source of high-quality protein.
- True
- False
- Unsure

11) Cow's milk contains the same types of protein as breast milk from women.
- True
- False
- Unsure

12) A calorie is a calorie, regardless if it comes from carbohydrate, protein, or fat.
- True
- False
- Unsure

Under Development

13) 4 ounces of chicken and 4 ounces of tofu have the same amount (grams) of protein.
- True
- False
- Unsure

14) Older adults (e.g. >50) have a relatively higher need for protein to maintain muscle mass later in life.
- True
- False
- Unsure

15) Strength athletes have an increased need for protein compared to the general population.
- True
- False
- Unsure

16) Endurance athletes have an increased need for protein compared to the general population.
- True
- False
- Unsure

17) Protein intake above the Recommended Dietary Allowance (RDA) is associated with an increased risk of developing certain types of cancer.
- True
- False
- Unsure

18) For healthy weight loss, it is important to consume more than the Recommended Dietary Allowance (RDA) of protein.
- True
- False
- Unsure

19) Dietary protein is primarily needed for the structure, function, and regulation of the human body's tissues and organs.
- True
- False
- Unsure

20) Dietary protein is important for a strong immune system, which protects the body from viruses and bacteria.
- True
- False
- Unsure

Under Development

21) A diet containing enough protein is important for bone health.
- True
- False
- Unsure

22) When it comes to maximizing human health, the source of protein is more important than the amount of protein consumed.
- True
- False
- Unsure

23) People following a diet higher in protein vs. processed carbohydrates tend to be less insulin resistant.
- True
- False
- Unsure

24) 8 ounces of plant-based milk (almond, cashew) and 8 ounces of cow's milk contain the same amount of protein.
- True
- False
- Unsure

25) Foods of animal origin are typically higher in protein than plant-based foods.
- True
- False
- Unsure

26) Abnormally high protein intake is associated with kidney and liver disease among the general population.
- True
- False
- Unsure

27) Pregnant women have an increased need for protein compared to the general population.
- True
- False
- Unsure

28) The building blocks of protein are amino acids.
- True
- False
- Unsure

Under Development

29) There are 9 amino acids that must be obtained from the diet.
- True
- False
- Unsure

30) All amino acids consumed in the diet are used to make protein in the human body.
- True
- False
- Unsure

31) Protein is a major component of bone health.
- True
- False
- Unsure

32) The main site of branched chain amino acid (BCAA), such as isoleucine, leucine, and valine, metabolism is in the skeletal muscle.
- True
- False
- Unsure

Attitudes

33) The impact of climate change can be reduced by consuming less meat, dairy products, and eggs.
- Strongly Agree
- Agree
- Undecided
- Disagree
- Strongly Disagree

34) Meat production is harmful to the environment.
- Strongly Agree
- Agree
- Undecided
- Disagree
- Strongly Disagree

35) Organic protein sources are better for the environment.
- Strongly Agree
- Agree
- Undecided
- Disagree
- Strongly Disagree

Under Development

36) Organic protein sources are healthier.

- Strongly Agree
- Agree
- Undecided
- Disagree
- Strongly Disagree

37) Plant-based protein is better for health than animal-based protein.

- Strongly Agree
- Agree
- Undecided
- Disagree
- Strongly Disagree

38) Protein consumption is essential to human health.

- Strongly Agree
- Agree
- Undecided
- Disagree
- Strongly Disagree

39) Egg consumption is harmful to human health.

- Strongly Agree
- Agree
- Undecided
- Disagree
- Strongly Disagree

40) The Recommended Dietary Allowance (RDA) for protein is adequate for healthy weight loss.

- Strongly Agree
- Agree
- Undecided
- Disagree
- Strongly Disagree

41) The Recommended Dietary Allowance (RDA) for protein is adequate for people following a vegetarian diet.

- Strongly Agree
- Agree
- Undecided
- Disagree
- Strongly Disagree

Under Development

42) Eating the same calorie level of either protein, carbohydrate, or fat has an equal effect on hunger.
- Strongly Agree
- Agree
- Undecided
- Disagree
- Strongly Disagree

43) Overeating dietary protein leads to the same gains in body fat as overeating carbohydrates or fat.
- Strongly Agree
- Agree
- Undecided
- Disagree
- Strongly Disagree

Behaviors

44) What is your primary source of nutrition information?
- Internet
- TV
- Books/Magazines
- Coach
- Physician
- Registered Dietitian
- Other (please specify)

45) How many people currently reside in your home, including yourself?
- 1 person
- 2-4 people
- More than 4 people

46) Are you the primary food purchaser and cook in your household?
- Yes
- No

47) What is the most important factor when shopping for a protein source?
- Price
- Taste
- Health
- Quality (high biological value)
- Other (please specify)

Under Development

48) How often do you eat away from home?

- Once a week
- Twice a week
- 3-4 times a week
- Every day

49) Are you a vegetarian or vegan?

- Yes
- No

50) Do you plan your meals around protein?

- Yes
- No

51) Approximately how many servings of protein (a serving is the size of a deck of cards) do you consume per day?

- None
- 1-3 servings
- 4-5 servings
- 6+ servings

52) When are you least likely to eat protein?

- Breakfast
- Lunch
- Snack
- Dinner
- Other (please specify)

53) What is your preferred source of protein?

- Lean meat (pork, beef, lamb, veal)
- Poultry (chicken, turkey)
- Fish/Seafood
- Dairy
- Eggs
- Plant-based (nuts and seeds, legumes/beans)
- Supplement

54) How many days per week do you consume high-quality protein (meat, eggs, milk, dairy)?

- None
- 1-3 days per week
- 4-5 days per week
- 6+ days per week

Under Development

55) If you consume high-quality protein, how many times per day?
- None
- 1-3 times per day
- 4-5 times per day
- 6+ times per day
- I do not eat animal protein

56) How often do you include a protein supplement (bars, shakes)?
- Every day
- 1-2 days per week
- A few days per month
- Never
- Other (please specify)

57) What is the biggest barrier that prevents you from consuming protein?
- No barriers
- Cost
- Convenience
- Dislike of protein taste/texture
- Unsure of how to prepare protein
- Too busy
- Other (please specify)

58) How many days per week do you participate in 30 minutes or more of structured exercise (walking, running, riding a bike)?
- None
- 1-3 days per week
- 4-5 days per week
- 6+ days per week

59) How many days per week do you participate in a structured strength training program (weight lifting)?
- None
- 1-3 days per week
- 4-5 days per week
- 6+ days per week

60) Which type of milk do you consume?
- Cow's milk
- Almond milk
- Soy milk
- Coconut milk
- None
- Other (please specify)

Under Development

61) If you consume cow's milk, which kind do you consume?

- Skim milk
- Low fat (1%) milk
- Reduced fat (2%) milk
- Whole milk
- I do not consume cow's milk

Attitudes Continued

62) Animal welfare should be protected.

- Strongly Agree
- Agree
- Undecided
- Disagree
- Strongly Disagree

63) Meat consumption is unhealthy.

- Strongly Agree
- Agree
- Undecided
- Disagree
- Strongly Disagree

64) Meat should not be consumed.

- Strongly Agree
- Agree
- Undecided
- Disagree
- Strongly Disagree

Under Development
